# Effects of Different Pediatric Drugs on the Color Stability of Various Restorative Materials Applicable in Pediatric Dentistry

**DOI:** 10.1155/2017/9684193

**Published:** 2017-01-10

**Authors:** Tamer Tüzüner, Sedanur Turgut, Ozgul Baygin, Nagehan Yilmaz, Elif Bahar Tuna, Bugra Ozen

**Affiliations:** ^1^Faculty of Dentistry, Department of Pediatric Dentistry, Karadeniz Technical University, Trabzon, Turkey; ^2^Faculty of Dentistry, Department of Prosthetic Dentistry, Karadeniz Technical University, Trabzon, Turkey; ^3^Faculty of Dentistry, Department of Pediatric Dentistry, Istanbul University, Istanbul, Turkey; ^4^Faculty of Dentistry, Department of Pediatric Dentistry, Istanbul Kemerburgaz University, Istanbul, Turkey

## Abstract

*Background*. The chronic recommendation of pediatric drugs could exhibit erosive and cariogenic problems.* Objective*. To evaluate the effects of different pediatric drugs on the color stability of various restorative materials.* Methods*. Five specimens (1 mm × 3 mm) were prepared and immersed in ten different pediatric drugs and agitated every 8 hours daily for 2 min up to 1 week. Between immersion periods, the samples were stored in artificial saliva. After 1-week period, Δ*E*^⁎^ values were calculated. Two-way ANOVA and Fisher's LSD test were used for statistical analysis at a level of *p* < 0.05.* Results*. Δ*E*^⁎^ values were only significantly influenced by restorative material factor (*p* < 0.001) and varied in the range of 2.08 and 6.55 units for all drugs/restorative materials. The highest Δ*E*^⁎^ was found in Ferrosanol B-composite (6.55 ± 1.38) and the lowest one was found in Dolven-glass ionomer (2.08 ± 0.40) pairwise. The most prominent Δ*E*^⁎^ value elevations were obtained in composite material compared to the compomer and/or glass ionomers in Macrol, Ferrosanol B, and Ventolin (*p* < 0.001; for all) and also for other drugs (*p* < 0.05). Dolven exhibited significantly higher values compared to Augmentin (*p* = 0.021), Macrol (*p* = 0.018), and Ventolin (*p* = 0.013) in compomer group.* Conclusion*. The clinically perceptible color changes for tested composite/pediatric drug pairwise can be more problematic than compomer and glass ionomers in pediatric dentistry.

## 1. Introduction

The demand for esthetic appearances in relation to various dental materials is increasing day by day in pediatric dentistry [1, 2]. Commonly, pediatric population could be treated with glass ionomers (GIC), compomers, and composites with the proper indications dependent upon the individual requirements of children [3–6].

Color changes have been considered as the major problems after using dental materials for a long period of time and these effects are mainly known as intrinsic and/or extrinsic factors which can be explained by the absorption and adsorption mechanisms [7–12]. Intrinsic factors have been shown as variations that occurred between resin matrix and filler components [7, 8]. Moreover, the staining effect of extrinsic factors has been indicated in the literature including various exogenous liquids/drinks in pediatric dentistry [2, 9–11, 13–16].

Pediatric liquid drugs are generally prescribed for children because of chronic requirements including analgesics, antibiotics, antihistaminics, antiepileptics, multivitamins, and antitussives [17–22]. Hence, the usage of these formulations in a short period may also be considered as prolonged outcomes [15, 21]. Their sugar containing properties might result in erosive and/or cariogenic potentials on teeth surfaces because of increased acidity [15, 17–23].

Even the previous studies have been focused on the acid degradation effects of pediatric medicines related to sucrose on tooth and restorative materials [15, 17–22, 24]; according to our knowledge, there is no much more evidence in which the staining effects of these formulations have been tested on dental materials applicable for pediatric dentistry.

Staining and discoloration have been commonly evaluated with devices such as spectrophotometry, colorimetry, or digital cameras [25–32]. The color values were reported in CIE *L*^*∗*^*a*^*∗*^*b*^*∗*^ system that enabled the evaluation of the degree of color change (Δ*E*^*∗*^) based on three coordinates. *L*^*∗*^ color coordinate represents lightness or brightness, *a*^*∗*^ represents greenness (positive) and redness (negative), and *b*^*∗*^ represents yellowness (positive) and blueness (negative). Numeric description of color permits precise definition of the magnitude of the color difference between objects [28, 29]. The color difference indicates whether a change in overall shade can be perceived by a human observer. Clinical color matching may be rated according to these Δ*E*^*∗*^ values and O'Brien [30] had reported that, based on clinical studies, the Δ*E*^*∗*^ values below 3.5 unit are unacceptable. Several studies [28, 29, 31] also considered the color differences greater than 3.5 between veneers and the teeth as clinically unacceptable.

So the aim of the present study was to assess the color changes of pediatric restorative materials after usage of some pediatric drugs for 1 week. Because of the above-mentioned details, the hypotheses of this study were considered as follows: (a) color values of the restorative materials would change and (b) drug types would influence the color stability of these materials.

## 2. Methods

The details of pediatric drugs and restorative materials used in this study were given in Tables 1 and 2.

### 2.1. Specimen Preparation

For each drug, *n* = 5 specimens (1 mm diameter and 3 mm in height) were prepared using molds. A total of 150 restorative (*n* = 50 for each) materials were prepared according to the manufacturers' instructions. Light-polymerized specimens (compomer and composite) were polymerized for 20 seconds using a LED (Elipar Free Light II, 3M/ESPE, St. Paul, MN, USA). After mixing the capsulated form of glass ionomer cements for 10 sec, they were left up to 5 minutes as for setting completion. Specimen surfaces were stabilized with Mylar strip band for providing smooth surfaces for all materials.

### 2.2. Color Change Measurement and Immersion Cycles

After the polymerization of restorative materials, the color measurements were done and recorded as baseline values, each specimen was rinsed with distilled water for 5 seconds, gently dried, and then immediately color values were obtained.

The color values (*L*^*∗*^, *a*^*∗*^, *b*^*∗*^) of each specimen were measured with a spectrophotometer (Vita EasyShade, Ivoclar Vivadent, Liechtenstein) in a viewing booth under a standard illuminant D65. Before the measurements, spectrophotometer was calibrated with its own special calibration tool and positioned in the center side of each specimen. Measurements were carried out according to the CIE *L*^*∗*^*a*^*∗*^*b*^*∗*^ system, 3 times for each sample, and the average was recorded. Afterward, they were (*n* = 5; for each material) immersed in 10 different 10 ml undiluted pediatric liquid in test tube and agitated every 8 hours daily for 2 min up to 1 week. Between immersion periods, the samples were stored in artificial saliva (sodium chloride (0.4 g/l), potassium chloride (0.4 g/l), calcium chloride-H_2_O (0.795 g/l), sodium dihydrogen phosphate-H_2_O (0.69 g/l), sodium sulphur-9H_2_O (0.005 g/l), and 1000 ml distilled water). After 1 week of cumulative immersion, second measurements for each materials were performed. Specimens were rinsed with distilled water for 5 seconds and gently brushed with a soft toothbrush for 15 seconds. At this point, color measurements were recorded with the same method, under the same conditions, and in the same manner as described for the baseline measurements and recorded as 1-week values. The calculation of the color variation Δ*E*^*∗*^ between two color positions (one week of storage and baseline) in 3-dimensional *L*^*∗*^*a*^*∗*^*b*^*∗*^ color space is as follows: color differences (Δ*E*^*∗*^) between the teeth of the same individual were calculated using the following formula: Δ**E**(**L**^**∗**^**a**^**∗**^**b**^**∗**^) = [(Δ**L**^**∗**^)^2^ + (Δ**a**^**∗**^)^2^ + (Δ**b**^**∗**^)^2^]^1/2^ in which

Δ**L**^**∗**^ is the difference between the *L*^*∗*^ values
Δ**a**^**∗**^ is the difference between the *a*^*∗*^ values
Δ**b**^**∗**^ is the difference between the *b*^*∗*^ values

### 2.3. Statistical Analysis

Statistical analyses were performed with SPSS for Windows 17.0. Shapiro–Wilk test was used to test the normality of Δ*E*^*∗*^ values. Two-way ANOVA and Fisher's LSD test were used for comparisons. The confidence level was set as 95%.

## 3. Results

Δ*E*^*∗*^ values varied in the range of averaged 2.08–6.55 for all drugs/restorative materials. The most prominent alteration was found in Ferrosanol B-composite (6.55 ± 1.38) and the most slight one was found in Dolven-glass ionomer (2.08 ± 0.40) pairwise (Table 4).

Two-way ANOVA revealed that Δ*E*^*∗*^ values were only significantly influenced by restorative material factor (*p* < 0.001) whereas no effects were found for pediatric drug (*p* = 0.491) and interactions (pediatric drug X restorative material) (*p* = 0.361) (Table 3).

Significantly higher Δ*E*^*∗*^ values were found as composite > compomer (*p* = 0.01) and composite > glass ionomer cement (*p* = 0.04) in Augmentin; composite > compomer/glass ionomer (*p* = 0.002) in Macrol; composite > glass ionomer cement (*p* = 0.01) and compomer > glass ionomer cement (*p* = 0.033) in Cefaks; composite > compomer (*p* = 0.011) and composite > glass ionomer (*p* < 0.001) in Calpol 6 plus; glass ionomer > composite (*p* = 0.003) and glass ionomer > compomer (*p*^a–c^ = 0.013) in Dolven; composite > compomer (*p* = 0.02) and composite > glass ionomer cement (*p* = 0.006) in Keppra; composite > compomer/glass ionomer (*p* < 0.001) in Ferrosanol B; composite > compomer/glass ionomer (*p* < 0.001) in Ventolin; composite > compomer (*p* = 0.009) and composite > glass ionomer cement (*p* = 0.014) in Sudafed. No differences were found in Atarax group (*p* > 0.05) (Table 4, for all).

Significantly higher Δ*E*^*∗*^ values were only found in compomer material for Dolven compared to the Augmentin (*p* = 0.021), Macrol (*p* = 0.018), and Ventolin (*p* = 0.013). Other pairwise comparisons did not exhibit any significant differences (*p* > 0.05) (Table 4; for all).

## 4. Discussions

Long-term and/or chronic usage of prescribed pediatric drugs could be problematic by decreasing plaque pH thus causing cariogenic and erosive potential in pediatric dentistry [17–24]. Moreover, prolonged color durability of pediatric dental restorative materials has been considered as one of the important factors included in esthetic requirements and much more visits to the dentist might also cause different problems such as increased costs of replacing the restorations and increased behavior management/dental anxiety issues. These have been found as another undesirable outcome [17–24].

Additionally, although many studies have evaluated the effects of lots of drinks and pediatric medicines with regard to erosion, caries, and/or color stability of teeth/dental materials [5, 7, 9, 10, 13–22, 24], according to our knowledge, the effects of pediatric drugs on the color stability of restorative materials have not been mentioned. Because of the above reasons, since the prescription is available for curing of pediatric age diseases by commonly used drugs, they have been tested in relation to the color stability of restorative materials.

Since the previous reports indicated that measuring quantitative color alterations by using CIE *L*^*∗*^*a*^*∗*^*b* with proper spectrophotometry and colorimetry techniques have several advantages such as repeatability, sensitivity, and objectivity, the spectrophotometry was used for calculating Δ*E*^*∗*^ values for each material/pediatric drug in this study [25, 26, 29, 31].

As is known, color changes might have been affected by the thickness and surface properties [10]. Since we only tested the color stability between baseline and 1-week period and provided smooth surfaces by using Mylar strip, we ignored calculating the effects of the above factors. Nevertheless, finishing the surfaces with standard techniques could also increase the discoloration resistance to the liquids [6, 7, 11, 27]. Thus, our Mylar strip method could be considered as a limitation factor in which the “resin-rich layer” was not properly removed. This might be further investigated.

The 1-week testing could be considered as a short period hence the children were usually recommended to take liquid drugs for ten days or one week, every eight hours [18]. Thus, present study was designed as suitable as possible to the clinical scenario. Moreover, it should be kept in mind that prolonged durations of taking medicines would be more problematic regarding the staining efficiencies of pediatric drugs.

The hypotheses of this study are both partially accepted. The most prominent alteration was found in Ferrosanol B-composite (6.55 ± 1.38) and the most slight one was found in Dolven-glass ionomer (2.08 ± 0.40) pairwise. Investigators reported that the GICs have been found as the most resistant material to staining related to their higher water content [7, 10]. Similarly, in the present study, GICs exhibited acceptable color stabilities compared with composite or compomer as well as for all tested pediatric drugs. However, color changes of resinous materials have been associated with chemical changes in the initiator system, activator, and water absorption of the monomers in composite. Additionally, the type/size of resin/filler particle and adsorption and absorption properties of the colourants are considered as the other detrimental factors for color stability [7, 8, 10, 11, 32]. Previous studies have investigated surface roughness and reported that GICs have rough surface, showing lower staining susceptibility relating to a lower water absorption rate or low resin content than surface textures [7, 11]. Although surface textures were not investigated in the present study, the highest color changes of composites can be attributed to the materials' ingredients.

Nevertheless, generally, the compomer restorations did not exhibit statistically significant discoloration and showed higher than 3.5 (>Δ*E*) values for some tested drugs. When comparing both resinous materials, compomer exhibited less serious color alterations compared to the composite which could also be related to glass filler particles inside the material as previously described [11]. However, all CIS-pediatric drug combinations resulted in below than 3.5 (<Δ*E*^*∗*^) which could clearly indicate that these materials usage with pediatric drugs can not cause perceptible clinical color changes as stated earlier [5, 7, 10, 11].

In the present study, Dolven caused statistically different staining compared to Augmentin (*p* = 0.021), Macrol (*p* = 0.018), and Ventolin (*p* = 0.013) for compomers. This may be related to the ingredients and other structural characteristics such as pH and amount of sugar of the staining agents as previously described [7, 11, 33]. Since the Ferrosanol-composite combination showed the worst staining outcomes, iron based syrups must be carefully prescribed because of the increased binding properties to the teeth surfaces [15].

Given the findings of present study, even though the artificial saliva was used to mimic oral conditions, exact staining problems for pediatric dental restorative materials could be influenced by taking the drugs in irregular intervals, composition and structural features of drugs, and individual salivary and fluid content differences with regard to buffering capacity [7, 8, 11, 15]. Thus, this in vitro model does not always mimic the real oral environmental conditions which can be reflected as a limitation factor. By the way, the lack of previous studies that tested the similar materials and methodology did not permit for an exact comparison with published literatures. Moreover, the various staining properties of tested pediatric drugs on the color stability of restorative dental materials possibly in relation to their pH levels and sugar contents should be kept in mind by pediatric health professionals and parents as similarly reported for possible long-term undesirable outcomes in terms of erosion and caries [15, 18, 19, 22, 23].

## 5. Conclusion

Within the limitations in this study, it can be concluded that

1. composites exhibited significant discoloration values when exposed to the commonly used pediatric drugs
2. glass ionomer cements seem to be more resistant to staining capacity of pediatric drug formulations
3. considering the resinous materials, compomers could exhibit less discoloration properties compared to the composites with different type of pediatric drugs
4. the information of the staining ability of commonly used pediatric drugs in combination with different restorative materials such as Ferrosanol B-composite and Dolven-compomer should be given to the pedodontists, pediatricians, and parents for alerting them to the risk of clinical discoloration
5. further studies need to be supported with in vivo study designs including commonly used drugs and restorative materials in pediatric dentistry

## Figures and Tables

**Table 1:** Pediatric liquid drugs (PLDs) used in the study.

PLDs	Generic name	Brand name
Antibiotics	Amoxicillin + clavulanic acid clarithromycin cephalosporins	Augmentin Macrol Cefaks
Analgesics	Paracetamol Ibuprofen	Calpol Dolven
Antiepileptics	Levetiracetam	Keppra
Multivitamins	Multivitamins	Ferrosanol B
Bronchodilator	Salbutamol	Ventolin
Anxiolytic	Hydroxyzine HCL	Atarax
Sympathomimetic	Pseudoephedrine HCL	Sudafed

**Table 2:** Restorative materials used in the study.

Product	Material type	Mixing	Curing	Manufacturer
Dyract XP	Polyacid modified composite resin	N/A	Light-cure for 20 seconds	Dentsply DeTrey, GmbH, Germany
Nova Compo-B Plus	Composite resin	N/A	Light-cure for 20 seconds	Imicryl Dental, Konya, Turkey
EQUIA	Glass ionomer cement	10 seconds with a mixer	Light-cure for 90 seconds	GC Corporation, Tokyo, Japan

**Table 3:** Two-way ANOVA interaction effects.

Variable	Sum of squares	df	Mean squares	*F*	*p*
Pediatric drug	23.704	9	2.634	0.943	0.491
Restorative material	298.904	2	149.452	53.508	0.000
Pediatric drug X restorative material	55.299	18	3.072	1.100	0.361
Error	335.168	120	2.793		
Total	3011.920	150			

df: degrees of freedom.

^*∗*^ *p* < 0.001; restorative material.

**Table 4:** Δ*E* values of tested restorative materials with pediatric drugs (mean ± sd).

Restorative materials	Composite	Compomer	Glass ionomer
	Δ*E* (mean ± sd)	Δ*E* (mean ± sd)	Δ*E* (mean ± sd)
Drug/brand name			
Augmentin	5.9 ± 2.12^a^	2.29 ± 0.92^B,b^	3.82 ± 1.61^c^
Macrol	5.63 ± 2.01^a^	2.23 ± 1.98^C,b^	2.34 ± 0.81^b^
Cefaks	6.40 ± 2.2^a^	4.67 ± 1.3^c^	2.4 ± 0.81^b^
Calpol 6 plus	6.64 ± 0.68^a^	3.90 ± 3.01^b^	2.57 ± 0.92^c^
Dolven	5.30 ± 1.45^b^	4.76 ± 3.41^A,c^	2.08 ± 0.40^a^
Keppra	5.68 ± 1.59^a^	3.18 ± 1.56^b^	2.72 ± 0.56^c^
Ferrosanol B	6.55 ± 1.38^a^	2.61 ± 2.03^b^	2.17 ± 0.69^b^
Ventolin	6.30 ± 1.76^a^	2.09 ± 1.2^D,b^	2.09 ± 0.48^b^
Atarax	4.14 ± 1.06	3.1 ± 2.46	2.41 ± 1.02
Sudafed	6.07 ± 1.84^a^	3.51 ± 2.34^b^	3.13 ± 1.13^c^

^*∗*^Significant differences were only found in compomer group between pediatric drug comparisons (*p*^A-B^ = 0.021; *p*^A–C^ = 0.018; *p*^A–D^ = 0.013); no differences were obtained in other pairwise comparisons *p* > 0.05.

^*∗*^Significant differences were found in Augmentin group; composite-compomer (*p*^a-b^ = 0.01) and composite-glass ionomer cement (*p*^a–c^ = 0.04), in Macrol group; between composite-compomer/glass ionomer (*p*^a-b^ = 0.002), in Cefaks group; composite-glass ionomer cement (*p*^a-b^ = 0.01) and compomer-glass ionomer cement (*p*^b-c^ = 0.033) in Calpol 6 plus group; between composite-compomer (*p*^a-b^ = 0.011) and composite-glass ionomer (*p*^a–c^ < 0.001), in Dolven group; between glass ionomer-composite (*p*^a-b^ = 0.003) and glass ionomer-compomer (*p*^a–c^ = 0.013), in Keppra group; composite-compomer (*p*^a-b^ = 0.02) and composite-glass ionomer cement (*p*^a–c^ = 0.006), in Ferrosanol B group; between composite- compomer/glass ionomer (*p*^a-b^ < 0.001) in Ventolin group; between composite-compomer/glass ionomer (*p*^a-b^ < 0.001) in Sudafed group; composite-compomer (*p*^a-b^ = 0.009) and composite-glass ionomer cement (*p*^a–c^ = 0.014). No differences were found in Atarax group (*p* > 0.05).
